# Sleep, circadian rhythms, and cognitive frailty in older Chinese immigrants (ROOTS Study)

**DOI:** 10.1002/alz70860_105744

**Published:** 2025-12-23

**Authors:** Ruixue Cai, Chenlu Gao, Lei Gao, Kun Hu, Peng Li

**Affiliations:** ^1^ Massachusetts General Hospital, Boston, MA, USA

## Abstract

**Background:**

Sleep and circadian health are critical for aging populations, influencing physical frailty and cognitive performance. Older adults who immigrate to the US to reunite with their families, known as zeroth‐generation immigrants, face unique challenges. Unlike first‐generation immigrants, their integration into American society is limited, and they are often reliant on their adult children for support. Despite evidence linking poor sleep health with frailty and cognitive decline, little research has explored these associations in zeroth‐generation immigrants. We aim to examine the relationships of sleep and circadian rhythms with frailty and cognition among older Chinese immigrants in the US.

**Method:**

We will recruit participants aged 65+ years old. Participants will complete tests and questionnaires, fitted for an actigraphy watch to wear for 10 consecutive days, and asked to finish a sleep diary of their sleep‐wake patterns during this period. Questionnaires will collect demographics, lifestyle, chronotype, living conditions, language experience, education and employment history, immigration and travel history, medical history and medication use, frailty, sleep quality, sleepiness, migration‐acculturative stress, depressive symptoms, and subjective cognitive decline. We will assess participants’ general cognitive abilities (Mini Mental State Exam), episodic memory (word list recall, story recall task), working memory (digit span test), and executive function (Trail making task, Stroop test). Physical assessments will collect height, weight, blood pressure, heart rate, grip strength, and walking speed.

**Result:**

We anticipate including 200 participants in the first phase of the study. Compared to first‐generation immigrants, zeroth‐generation participants are expected to report poorer sleep and circadian health, including lower sleep quality, shorter sleep duration, increased daytime sleepiness, and greater rest‐activity rhythm disturbances. We expect higher frailty prevalence and worse cognitive performance among zeroth‐generation immigrants, with acculturative stress contributing to these disparities. Additionally, we expect stronger associations between poor sleep health, frailty, and cognitive impairments in zeroth‐generation immigrants, mediated by heightened acculturative stress.

**Conclusion:**

This study will provide new insights into the sleep and circadian health challenges faced by zeroth‐generation Chinese immigrants and their impact on frailty and cognitive aging. Findings will inform interventions and policies to promote health equity, addressing the specific needs of this vulnerable demographic.

